# Changes in microflora in dental plaque from cancer patients undergoing chemotherapy and the relationship of these changes with mucositis: A pilot study

**DOI:** 10.4317/medoral.19934

**Published:** 2015-02-07

**Authors:** Iole Vozza, Vito Caldarazzo, Livia Ottolenghi

**Affiliations:** 1 DDS, PhD Department of Oral and Maxillo-facial Sciences, Sapienza University of Rome; 2 MD, DDS Department of Oral and Maxillo-facial Sciences, Sapienza University of Rome; 3 DDS Department of Oral and Maxillo-facial Sciences, Sapienza University of Rome

## Abstract

**Background:**

To assess changes in oral micro flora in dental plaque from cancer patients within 7 days of the first course of chemotherapy, and the relationship of the changes with mucositis.

**Material and Methods:**

Thirty cancer patients, divided into a test group undergoing chemotherapy and a control group no undergoing chemotherapy, were enrolled in this pilot study. Oral micro flora were cultured from three samples of dental plaque at t0 (before chemotherapy), t1 (1 day after chemotherapy) and t2 (7 days after chemotherapy). Single and crossed descriptive analyses were used to establish prevalence, and the χ2 test was used to establish the statistical significance of the differences observed in distributions (significance level: *P*<0.05.

**Results:**

In most patients (57%), oral micro flora consisted mainly of Gram-positive cocci, while the remaining 43% of the bacterial flora also had periodontal-pathogenic species. No *Porphyromonas gingivalis* appeared in the test group. *Actinobacillus* was the least frequently found bacterium among periodontal pathogens in the test group, while *Fusobacterium nucleatum* was the most frequently found. No significant differences were found in quantitative bacterial changes between t0, t1 and t2 in either the test or control groups, or between the two groups. According to World Health Organization scores, oral mucositis developed in 10 patients (66.6%) in the test group.

**Conclusions:**

The results of this pilot study indicate that there were no changes in microflora in dental plaque in cancer patients within 7 days of the first course of chemotherapy. No correlations between oral mucositis and specific microorganisms were assessed.

**Key words:** Oral microflora, dental plaque, cancer patients, chemotherapy.

## Introduction

The human oral cavity is inhabited by upwards of 500 species of bacteria (1), most of which are harmless com mensal organisms. Others, however, are pathogenic and are involved in the development of dental caries, periodontal diseases, and acute or chronic infections.

Cytotoxic chemotherapy compromises the oral defense mechanisms, either by direct mucosal damage or by neutropenia, potentially causing an overall shift in oral micro flora. In patients undergoing chemotherapy, there is an increase in the number and proportion of some bacteria associated with periodontal diseases (*Actinobacillus actinomycetemcomitans, Porphyromonas gingivalis and Fusobacterium nucleatum*) (2-4), along with a diminished effectiveness of immune defense against infection (5). Mucositis is an oral complication that affects 30–40% of patients receiving chemotherapy and radiotherapy and 80% of those undergoing hematopoietic stem cell transplantation. It is a multi factorial disease defined as epithelial thinning associated with intense erythema, ulceration, pain, bleeding and increased risk of infection (6). The cytotoxic effects of anticancer drugs against high-turnover tissues such as the oral epithelium, and the local effects of radiation on the oral mucosa are responsible for this event, which compromises quality of life and may interfere with management of the disease. Mucositis typically appears between 7 and 14 days after the initiation of chemotherapy, and is usually preceded by a subjective complaint of soreness or a burning sensation. Drugs most likely to cause mucositis include doxorubicin, bleomycin, fluorouracil, and methotrexate (7).

It is thought that the incidence and severity of cancer-chemotherapy-associated mucositis is caused in part by changes in the oral bacterial micro flora. Oral microorganisms are believed to be involved in the ulceration phase, where they probably intensify the inflammatory process and aggravate or promote the formation of ulcers (8). However, until now, it has been unclear whether there is an association between periodontal pathogens and mucositis (2).

Given the significant impact of oral mucositis on quality of life, it is essential to try to prevent it by all means possible. Currently, there are several treatments, but none of them has been validated in definitively (9). The aim of this pilot study was to assess changes in oral micro flora in dental plaque in cancer patients within 7 days from the first course of chemotherapy, and their relationship with mucositis.

## Material and Methods

- Patients

We enrolled 30 patients (16 men and 14 women, aged 32–59 years) with solid malignancy. who had no previous adjuvant radiotherapy or recent anti microbial or anti viral treatment. The primary, stage II, squamocellular cancer was located in the lungs (5 men and 1 woman), colon–rectum (8 men and 5 women patients), prostate (3 men) and breast (8 women). The study was conducted at the Oncology Unit, Fiorini Hospital, Terracina, Italy. The study conformed to the Helsinki Declaration and the study was approved by the Medical Ethical Committee of Sapienza University of Rome. All patients gave their written informed consent. The patients were divided in two groups of 15: the test group consisted of patients undergoing a first course of chemotherapy with docetaxel or 5-fluorouracil and oxaliplatin; the control group consisted of patients not undergoing chemotherapy because of the stage of their disease and because they did not have adequate numbers of platelets and leukocytes ( Table 1).

**Table 1:**
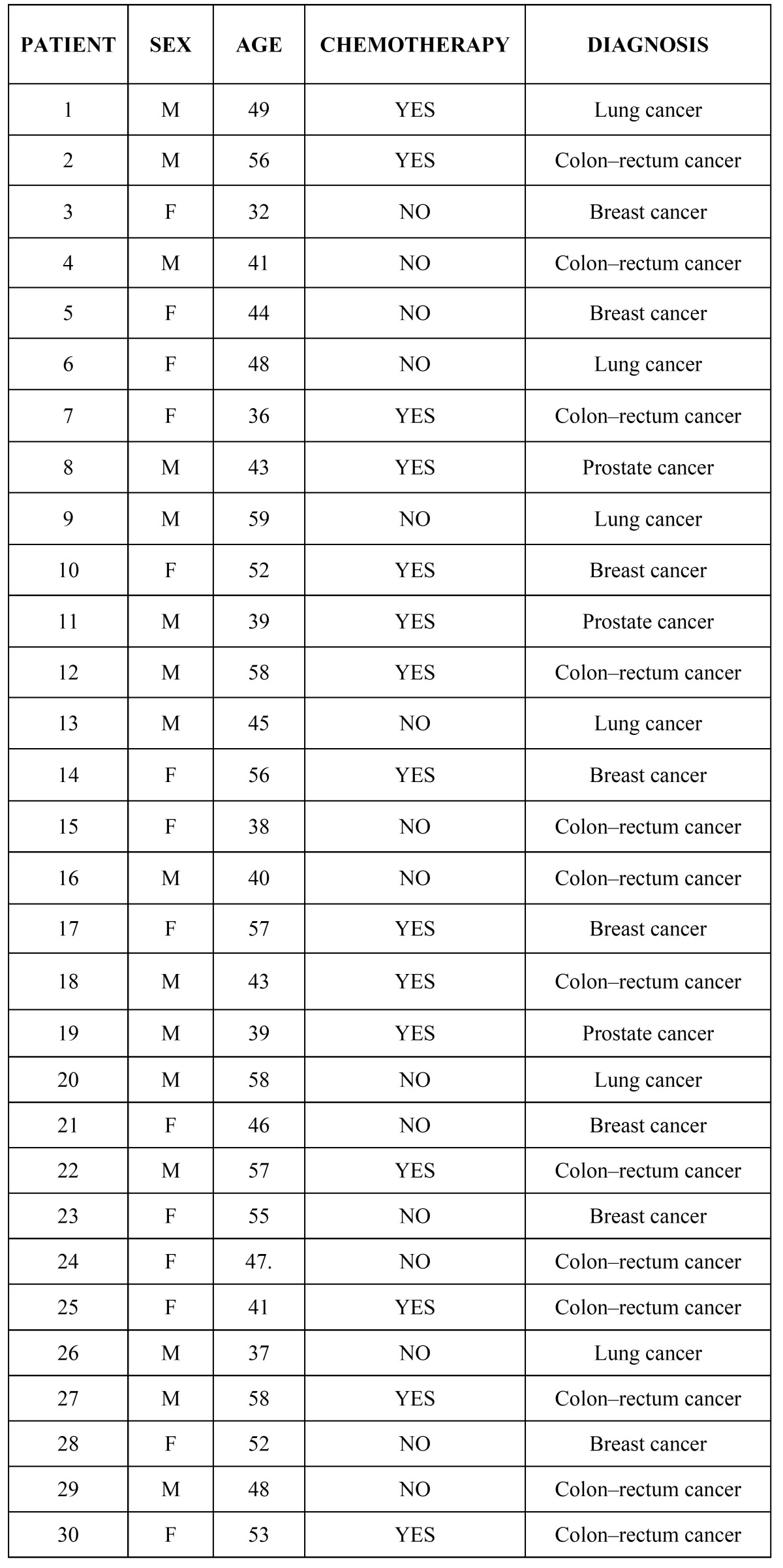
Patient characteristics.

- Microbial analysis

Oral mucositis was scored according to World Health Organization (WHO) criteria (10) at eight nonkeratinized anatomical sites (labial and buccal mucosa, lateral and ventral tongue, floor of mouth, and soft palate) by one trained dentist (V.C.). Oral micro flora were cultured from plaque specimens. All patients were sampled at time zero (t0) (immediately before chemotherapy), and on t1 (1 day after infusion) and t2 (7 days after infusion). Control subjects were sampled on equivalent dates. Sampling was done at the same time of day, approximately 2 h after breakfast. For each individual, the supra gingival plaque of the right lower premolars was collected with a sterile swab. All specimens were processed within the following 4 h. Following serial dilution, 100 µl of each dilution was plated on Schaedler Selective Blood Agar plates supplemented with 5% bovine blood (Biolife Italiana, Milan, Italy) and incubated in 80% nitrogen/10% hydrogen/10% CO2 at 35°C to monitor *P. gingivalis*, *F. nucleatum*, *Actinobacillus* spp. and *Peptostreptococcus* micros. An additional 100 μl was plated on Columbia agar containing 5% bovine blood (Biolife Italiana) in 5% CO2 to monitor *Gemella* spp., *Streptococcus* spp., *Leuconostoc* spp., and *Granulicatella* spp. Microorganisms were identified by standard procedures (11) as well as the production of a set of metabolic enzymes (as tested with Rapid ID 32A and Rapid ID32 Strep) (12,13). With regard to bacterial counts, the results were expressed in MCF, equivalent to 1.5×108 cells/ml.

- Statistical analysis

Single and crossed descriptive analyses were used to establish prevalence, and the χ2 test was used to establish the statistical significance of the differences in distributions (significance level: *P*<0.05). The data were analyzed using SPSS statistical software.

## Results

**Figure 1:**
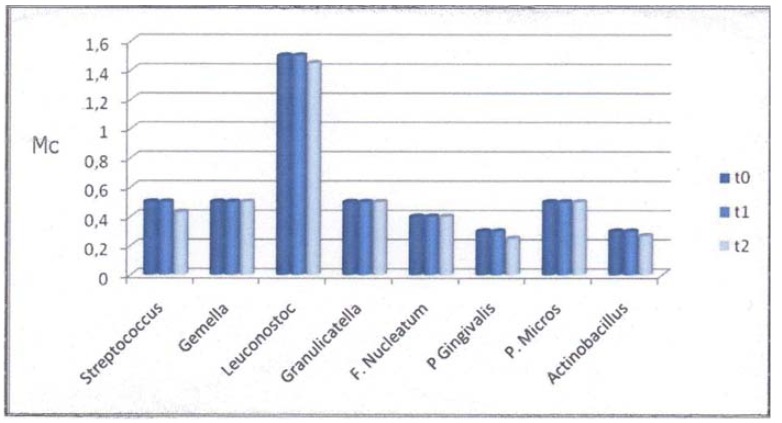
Mean numbers of bacteria in the samples in the test group.

**Figure 2:**
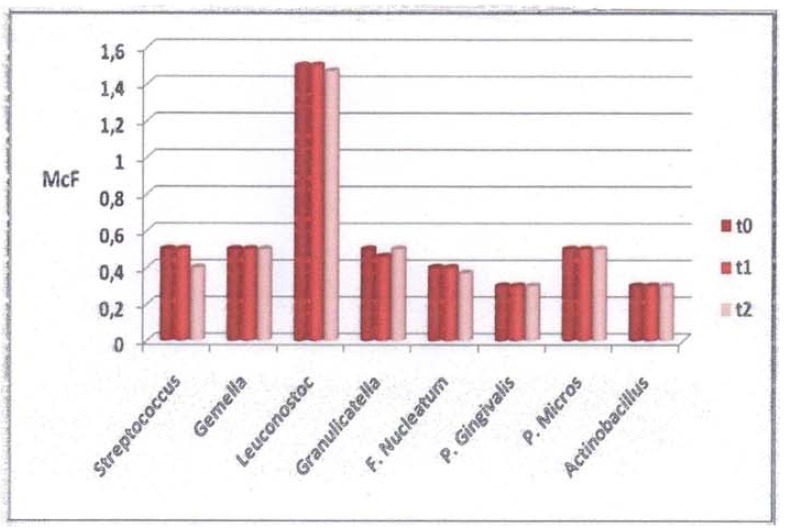
Mean numbers of bacteria in the samples in the control group.

**Figure 3:**
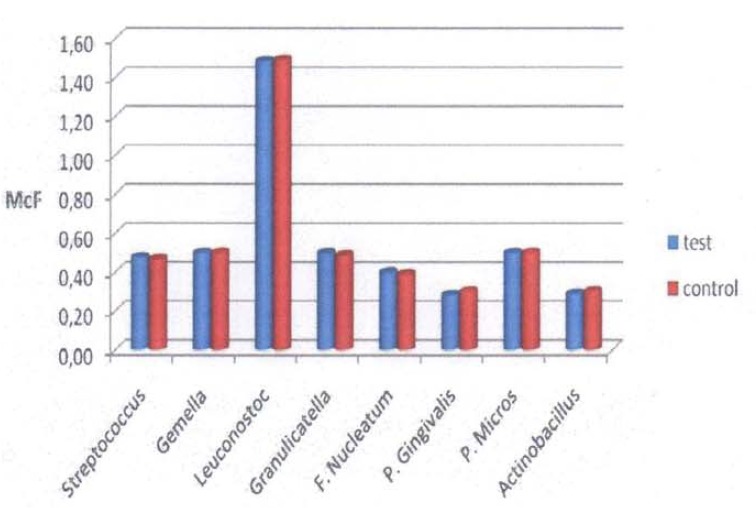
Cross-sectional analysis of mean bacterial counts between the two groups.

Oral mucositis, according to WHO scores, involving nonkeratinized sites developed in 10 patients (66.6%) in the test group: eight with grade 1 and two with grade 2. No ulcerations on the keratinized mucosa were scored. No mucositis developed in the control group.  Table 2 shows 17 patients (57%) who developed plaque that consisted predominantly of saprophytic Gram-positive cocci (*Streptococcus* spp., *Leuconostoc* spp., *Granulicatella* spp. and *Gemella* spp.). Nine of these patients underwent chemotherapy (53%). The other 13 patients (43%) developed periodontal pathogens (*F. nucleatum*, *P. gingivalis*, *Actinobacillus* spp. and *Pep. micros*). Six of these patients (46%) were undergoing chemotherapy. No *P. gingivalis* appeared in the test group. *Actinobacillus* spp. were the least frequently found periodontal pathogen in the test group (6.6%), while *F. nucleatum* was the most frequently found (20%). No significant differences were found in bacterial changes between t0, t1, and t2 in the test group (Fig. 1). In the control group, the bacterial count remained unchanged during the observation period (Fig. 2). At t0, t1 and t2, differences in qualitative and quantitative variations between the two groups were not significant (Fig. 3).

**Table 2:**
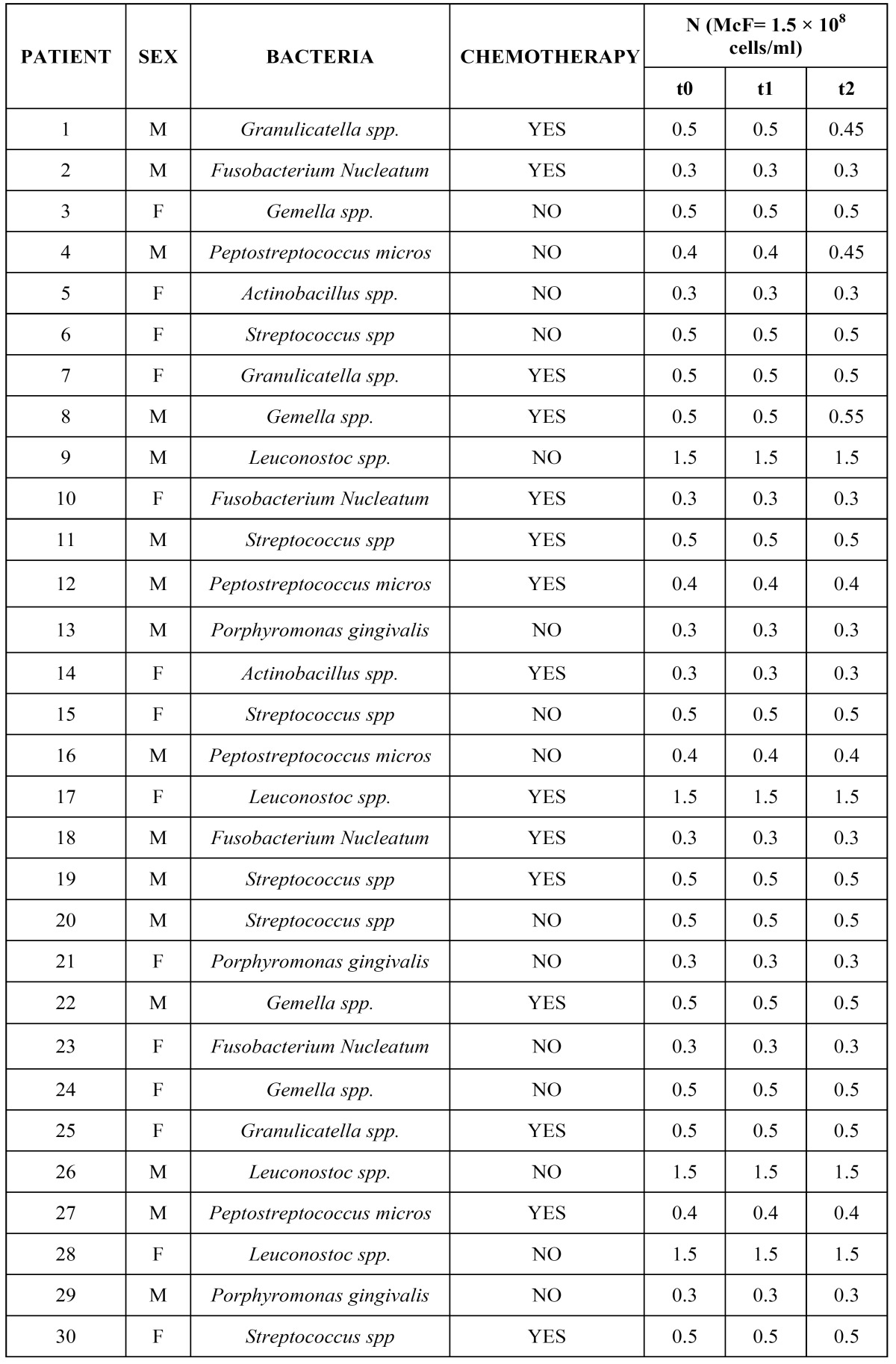
Mean numbers of bacteria in the study population.

## Discussion

Supra gingival plaque is influenced by saliva and gingival fluid and allows the growth of aerobic and anaerobic organisms (14), ultimately leading to complex micro flora dominated by Gram-positive bacteria, particularly streptococci. This flora can be representative of the oral flora during chemotherapy (15), as found in our present study. The micro flora undergo modifications during the day, particularly due to eating, and for this reason, all sampling was done 2 h after breakfast. The standardization of sampling allowed us to minimize variations related to this parameter.

The microorganisms monitored in this study were saprophytic species of the oral cavity (*Streptococcus* spp., *Leuconostoc* spp., *Granulicatella* spp. and *Gemella* spp.) and species associated with periodontal pathology (*P. gingivalis*, *Actinobacillus* spp., *Peptostreptococcus* spp. and *F. nucleatum*). These periodontal pathogens are known for their association with periodontal diseases in immunosuppressed individuals (15-17). In our pilot study *F. nucleatum* was the most frequently found periodontal pathogen in dental plaque of patients undergoing chemotherapy. However, none of our patients showed any sign of serious periodontal pathology and periodontal probing, was not part of the standard of care during our study. Therefore, it was not possible to make such conclusions from this study.

The dental plaque flora are constantly influenced by external sources, such as nosocomial infections, gastroesophageal reflux, and systemic and oral treatments. Topical, oral and parenteral anti microbials before and during cancer chemotherapy should alter the quantitative and qualitative oral micro flora profile (18). For this reason, the use of anti microbial agents was an exclusion criterion for our study. Children differ from adults in their oral micro flora, and in their response to chemotherapeutic regimens. Most of the oral bacterial changes noted in pediatric studies involved Gram-positive streptococci and staphylococci, whereas in studies of adults, most changes involved Gram-negative organisms such as Enterobacteriaceae and *Pseudomonas* spp. (15).

There is no consensus regarding qualitative and quantitative changes in oral micro flora during cancer chemotherapy, or a clear pattern or association between mucositis and changes in oral micro flora (2). Previous studies have differed in many important aspects, including patient populations and presence of a control group, chemotherapeutic regimens, use of anti micro bialsduring chemotherapy, sample sites and number of samples collected, collection times and methods, microorganisms cultured, and the scoring method for mucositis. Thus, it is difficult to compare our results to those of other studies. Our results showed that, although there was a reduction in the number of oral bacteria in 5% of patients in the test group, in the remaining 95%, there was no significant change in the number of bacteria analyzed from t0 to t2. Similarly, the test group showed no change in bacterial micro flora between beginning chemotherapy and at the end of treatment.

The cross-sectional analysis showed no significant differences between the test and control groups. In slightly more than half of the patients (57%), the oral micro flora consisted mainly of Gram-positive cocci (saprophytic species of the oral cavity), while the remaining 43% of the patients had bacterial flora that also had periodontal-pathogenic species. The only difference between the two groups was the incidence of mucositis, which was present only in the test group. These results suggest that bacterial pathogenicity is due less to changes in the intrinsic micro-habitat of the oral cavity, and more to a decrease in the efficiency of the immune response (19). However in this study, the relationship between leukocyte counts and quantitative oral micro flora changes was not determined.

The combination of mucositis and granulocytopenia increases the risk of systemic infection resulting from invasion of oral micro flora into the bloodstream. However, although it is postulated that some oral bacteria may exacerbate mucositis, it cannot be determined from the results that the presence of local or systemic bacterial infection correlates with the onset and severity of mucositis (20). *P. gingivalis* was consistently associated with oral ulcerations in a study of hematopoietic stem cell transplant patients and had a positive predictive value (8). *P. gingivalis* possesses several virulence factors such as fimbriae that enable the bacterium to at) ach to and invade epithelial cells (21), and a lipopolysaccharide capsule that is highly antigenic and can induce the production of proinflammatory cytokines (22). These virulence factors might prolong or intensify oral ulcerations and could explain the role of *P. gingivalis* in mucositis. Nevertheless, in our study, no patient undergoing chemotherapy had *P. gingivalis* in the plaque samples.

## Conclusion

Within the limitations of the small sample size of our pilot study, it can be concluded that no changes occur in micro flora in dental plaque in cancer patients within 7 days from the first course of chemotherapy. No correlations between oral mucositis and specific microorganisms were assessed. More patients are required to increase the reliability of the results, and more detailed studies are necessary to understand the relationship between chemotherapy, alterations in the nature and magnitude of the oral micro flora, and the presence of mucositis. Better characterization of changes in oral micro flora would be obtained using molecular biological techniques. This would help our understanding of the potential role of oral micro flora in the development and exacerbation of oral mucositis. Data from such work could be directed toward developing and testing selective anti microbial therapies for the prevention and management of mucositis during cancer chemotherapy.
